# A Study of the Structural and Surface Morphology and Photoluminescence of Ni-Doped AlN Thin Films Grown by Co-Sputtering

**DOI:** 10.3390/nano12213919

**Published:** 2022-11-07

**Authors:** Mohsin Khan, Ghazi Aman Nowsherwan, Aqeel Ahmed Shah, Saira Riaz, Muhammad Riaz, Ali Dad Chandio, Abdul Karim Shah, Iftikhar Ahmed Channa, Syed Sajjad Hussain, Rashid Ali, Shahzad Naseem, Muhammad Ali Shar, Abdulaziz Alhazaa

**Affiliations:** 1 Centre of Excellence in Solid State Physics, University of the Punjab, Lahore 54590, Pakistan; 2 Wet Chemistry Laboratory, Department of Metallurgical Engineering, NED University of Engineering and Technology, University Road, Karachi 75270, Pakistan; 3 Department of Chemical Engineering, Dawood University of Engineering and Technology, Karachi 74800, Pakistan; 4 Department of Materials Science and Engineering, Ghulam Ishaq Khan Institute of Engineering Sciences and Technology, Topi 23640, Pakistan; 5 Department of Mechanical & Energy Systems Engineering, Faculty of Engineering and Informatics, University of Bradford, Bradford BD7 1DP, UK; 6 Department of Physics and Astronomy, College of Science, King Saud University, Riyadh 11451, Saudi Arabia

**Keywords:** nickel, AlN, co-sputtering, doping

## Abstract

Aluminum nitride (AlN) is a semiconductor material possessing a hexagonal wurtzite crystal structure with a large band gap of 6.2 eV. AlN thin films have several potential applications and areas for study, particularly in optoelectronics. This research study focused on the preparation of Ni-doped AlN thin films by using DC and RF magnetron sputtering for optoelectronic applications. Additionally, a comparative analysis was also carried out on the as-deposited and annealed thin films. Several spectroscopy and microscopy techniques were considered for the characterization of structural (X-ray diffraction), morphological (SEM), chemical bonding (FTIR), and emission (PL spectroscopy) properties. The XRD results show that the thin films have an oriented c-axis hexagonal structure. SEM analysis validated the granular-like morphology of the deposited sample, and FTIR results confirm the presence of chemical bonding in deposited thin films. The photoluminescence (PL) emission spectra exhibit different peaks in the visible region when excited at different wavelengths. A sharp and intense photoluminescence peak was observed at 426 nm in the violet-blue region, which can be attributed to inter-band transitions due to the incorporation of Ni in AlN. Most of the peaks in the PL spectra occurred due to direct-band recombination and indirect impurity-band recombination. After annealing, the intensity of all observed peaks increases drastically due to the development of new phases, resulting in a decrease in defects and a corresponding increase in the crystallinity of the thin film. The observed structural, morphological, and photoluminescence results suggest that Ni: AlN is a promising candidate to be used in optoelectronics applications, specifically in photovoltaic devices and lasers.

## 1. Introduction

Recently, many scientific and technological efforts have been made, focusing on improving the physical properties of AIN, such as adding catalysts [1], doping metal and metal oxides [2,3,4], preparing composite materials [5], etc. Among these methods, doping metal materials into AIN has been proven to be a simple and efficient approach to enhance its physical and chemical properties. Suitable dopants are often mixed into the AlN matrix which modifies its microstructure and defects, resulting in changes in its optical, physical, and chemical properties.

The researchers used room-temperature ferromagnetic substances in dopant materials, such as GaN, ZnO, and AlN. The incorporation of transition metals (i.e., Mn, Ni, Fe, Cu, and Cr) in ferromagnetic materials brings promising improvements in magnetic, electrical, and optical properties. Over the last decade, researchers showed great interest in AlN due to its extraordinary physical, optical, chemical, and structural properties, such as a high thermal conductivity of 3.85 W/cm K, high transmission, wurtzite crystal structure, and a very high direct bandgap of 6.12 eV at room temperature [6,7,8]. It has a melting point of around 2473.15 K and is a highly stable material in inert atmospheres at high temperatures. AlN semiconductors are extensively utilized in various fields of science and technology, such as modern storage devices [9], the petroleum industry [10], electroluminescent devices [11], photolithography [12], and optoelectronics [13]. Furthermore, these semiconductors are also utilized as dielectric layers in optical storage media, and electronic substrate sterilization. Optical properties such as emission [14], resistivity [15], bandgap [16], reflectance [17], and transmittance will all be affected by introducing transition and rare-earth metals as impurities to the AlN matrix. Lanthanides with an oxidation number of +3 are compatible with III-V semiconductors and may be doped into the matrix of these materials. For the past two decades, it has been considered as a preferred material for radio frequency (RF) bandpass filters. Holmium (Ho)-doped AlN thin films [18] showed significant improvements in optical, electrical, and magnetic properties after irradiation. The fascinating and useful phenomenon associated with Ni-doped phosphors is broad near-infrared emission (NIR). Ni^2+^ ions are reported to give ultra-wide NIR emission when doped in a suitable host lattice. These Ni-doped phosphors are found to have promising applications for tunable lasers and wideband optical amplifiers. Ni^2+^ ions can be incorporated in many tetrahedral crystal structures as activators, but crystal environment and field strength may cause variations in energy levels and energy gaps. So, luminescence can be modified by precisely changing the crystal field strength [19,20]. For the manufacture of devices based on AlN thin films, a deeper knowledge of mechanical properties is required, since contact loading during manufacturing or packing may drastically affect the performance of these devices [21,22]. As a result, the necessity to research mechanical characteristics of materials for device applications, particularly in nanotechnology, is growing.

Trivalent transition metals, such those found in rare-earth deposited III-nitride hosts, generate stable luminescence centers, and exhibit high luminescence efficiency in III-V materials. Transition metals, in contrast to rare earth metals, can produce multiple-charge ions at will, and the energy levels of these ions are profoundly influenced by the local field from the semiconductor host [23,24]. The luminescence properties of lanthanide-activated films should be considerably different from those of transition-metal-incorporated III-V hosts. Metal-doped AlN films can be synthesized by different techniques such as sol gel [25], pulsed laser deposition (PLD), chemical vapor deposition (CVD) [26], molecular beam epitaxial (MBE) [27], spray pyrolysis [28], DC/RF sputtering [29,30] etc. Among these techniques, RF and DC sputtering have attracted significant interest due to their many advantages, such as high deposition rates, low substrate temperatures, easy control over the composition of deposited films, low costs, and uniformity over large areas of the substrates. However, high-crystalline-quality metal-doped AlN thin films were usually fabricated by sputtering with high substrate temperatures or thermal annealing [31]. There is a growing need to investigate the preparation and characterization of high-crystalline-quality Ni: AlN films deposited by RF sputtering at room temperature, as films deposited at low temperatures demand lower consumption and a shorter cooling time, which would reduce the cost of production and improve the productivity, but few studies have covered this.

In the current study, Ni-doped AlN films were produced at room temperature using both DC and RF sputtering. The primary goal of this research was to investigate the structural, morphological, photoluminescence, and optical aspects of thin film deposition. The goal of annealing and incorporating Ni into AlN was to improve structural, morphological, and photoluminescence properties to create a promising material for optoelectronic applications.

## 2. Experimental Details

**Figure 1:**
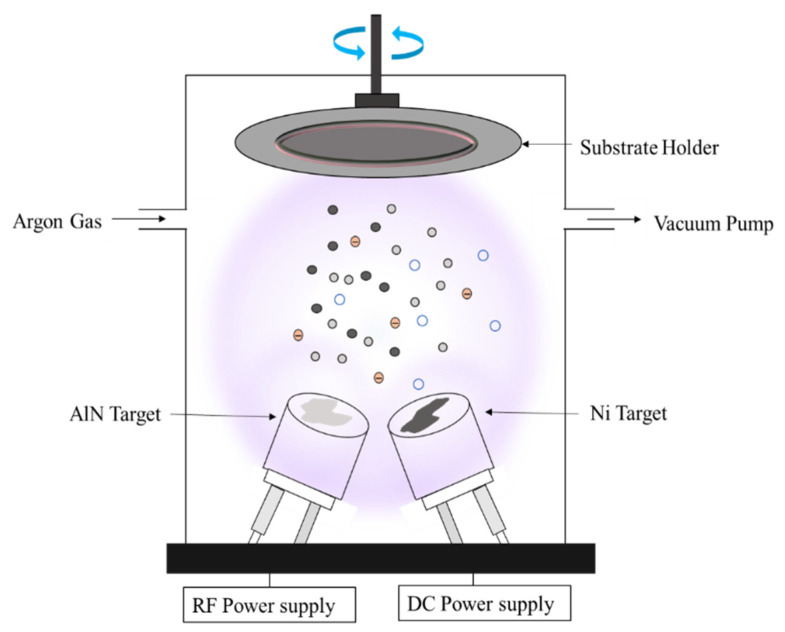
Schematic illustration of co-sputtering.

The Ni-doped AlN thin films were deposited using the dual-magnetron sputtering assembly, as shown in Figure 1. The co-sputtering of AlN and Ni was accomplished using a combined RF and DC sputter system, where the AlN target exhibits 99.9% purity and the Ni target has 99.95% purity. The thickness and diameter of the target were 3 mm and 2 inches, respectively. For comparative analysis and characterization purposes, both silicon and copper wafers were used as substrates during the sputtering process. Before deposition, the substrate was carefully washed with acetone and isopropyl alcohol (IPA). It was then cleaned in an ultrasonic bath for several minutes to remove residuals and organic contaminants, and nitrogen was finally sprayed over the slide to remove any leftover impurities and sticky particles. To begin with, we employed argon gas at a flow rate of 50 sccm and a chamber operating voltage of 300 V for 5 min to clean the chamber using the plasma-cleaning method. Following that, we placed the substrate on the substrate holder, with a gap of 60 mm between the substrate and the target. Ni sputtering was accomplished with the aid of DC sputtering. A DC power of 20 W was supplied, which drew a current of 74 mA and a voltage of 281 V during deposition, and the base pressure was held at 6.6 × 10^−3^ Pa. The substrate was spanned at 100 revolutions per minute. While RF sputtering was employed to deposit AlN, a high vacuum was achieved with the help of a turbo molecular pump, which rotated at a speed of 12,000 rpm. The working pressure for RF sputtering was 2.4 Pa, the RF power supply was 120 W, and the operating voltage of RF supply was 100 V. The argon gas flow rate was 85 sccm. Ni was doped by giving the Ni target less time to sputter than the AlN target. The deposition time evaluated for Ni was 5 min, while AlN took 40 min.

## 3. Annealing Process

**Figure 2:**
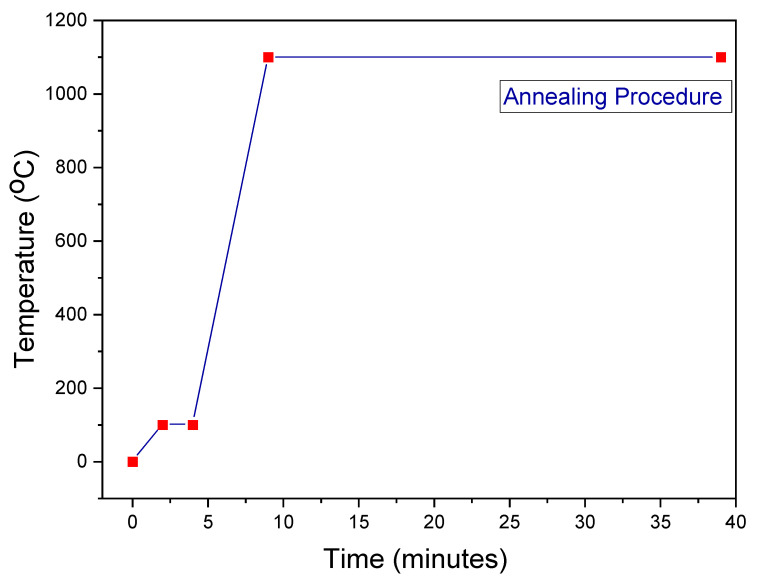
The annealing process.

The deposited thin films were annealed in a Noberthum furnace at 200 mbar in an N_2_ gas atmosphere. After the furnace was turned on, it reached a temperature of 100 °C in 2 min and remained there for another 2 min. It reached 1100 °C in 5 min and maintained that temperature for 30 min. Afterwards, the furnace was deactivated. We removed the sample from the furnace after nearly 5 h of waiting once it reached room temperature. Figure 2 graphically illustrates the annealing procedure.

## 4. Characterization of Samples

The structure, surface morphology, chemical bonding, and photoluminescence characteristics were studied using several spectroscopic and microscopy techniques (i.e., XRD, SEM, FTIR, and PL spectroscopy). Cu Kα radiation with a wavelength of 1.5406 nm was utilized to analyze X-ray diffraction data acquired with the Bruker D8 Advance. A morphology of deposited thin films was acquired using a Jeol (Tokyo, Japan) JSM-6380A scanning electron microscope. Agilent Cary 630 was used to perform the FTIR analysis for the purpose of element identification. Using an FS5 spectrofluorometer, the photoluminescence (PL) properties of nickel-doped aluminum nitride thin films (as-deposited and annealed) were also examined.

## 5. Structural Properties

**Figure 3:**
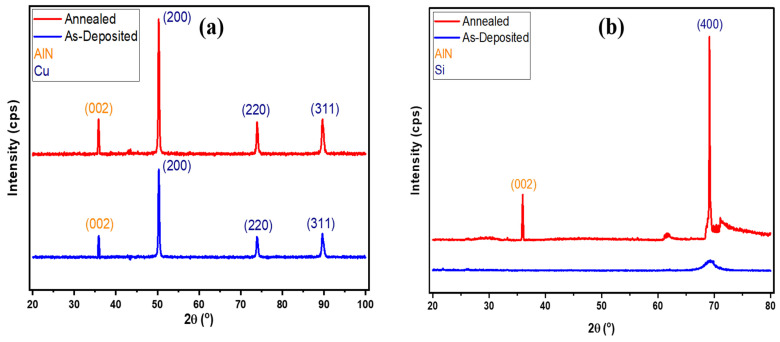
X-ray diffraction (XRD) patterns of the deposited Ni-doped AlN thin film with indexed lattice planes (**a**) Copper substrate and (**b**) Silicon substrate.

Structural studies of as-deposited and annealed Ni-doped AlN thin film were carried out using the Bruker D8 discover diffractometer instrument and Cu Kα X-rays with a wavelength of 1.5406 Å. The data were collected over the angular range 2θ of 20° to 100° and 80° with the collection step size of 0.02° and a collection time of 1 s per step. The data were indexed using the analytical method, which drew comparisons with the standard reference pattern in the library of Xpert High Score. The XRD patterns of the as-deposited and annealed thin films on different substrates are presented in Figure 3.

Figure 3a shows the XRD pattern of Ni-doped AlN thin films formed on a Cu substrate. It is found that after annealing, the corresponding peak positions for grown thin films were not affected. However, the peak intensity was enhanced due to an improvement in defects and an enhancement in grain size. The presence of sharp and narrow diffraction peaks indicates the strong crystallinity of an AlN thin film, in which Ni atoms were successfully replaced at atomic substitutional sites and produced on a Cu substrate. The medium intensity peak at 35.77° was caused by the existence of an AlN (002) plane in the sputtered thin film, as confirmed by JCPDS card 00-025-1133 [31,32]. Other peaks, i.e., (200), (220), and (311), were also confirmed by JCPDS card 00-001-1241, attributable to the Cu substrate.

The XRD pattern of Ni-doped AlN thin films formed on the as-deposited and annealed Si substrate is shown in Figure 3b. It is analyzed that there were no significant peaks in the as-deposited thin films, which validates the amorphous nature of deposited thin films. After annealing, two peaks were formed, one due to AlN and the other due to the Si substrate. The medium intensity peak at 35.77° was caused by the presence of an AlN (002) plane in the sputtered thin film, as confirmed by JCPDS card 00-025-1133. Another (400) peak was attributed to the Si substrate validated by JCPDS card 01-080-0018 [33]. The observed results suggest that the deposited thin film had a c-axis-oriented hexagonal wurtzite crystal structure.

The average grain sizes, calculated using the Scherrer formula [34,35], were about 340 Å and 442 Å on the Cu and Si substrates, respectively.

$$D=\frac{K\lambda }{\beta \;\cos\theta }$$

Further crystallographic constants of crystal structure are provided in Table 1.

## 6. Surface Morphology

**Figure 4:**
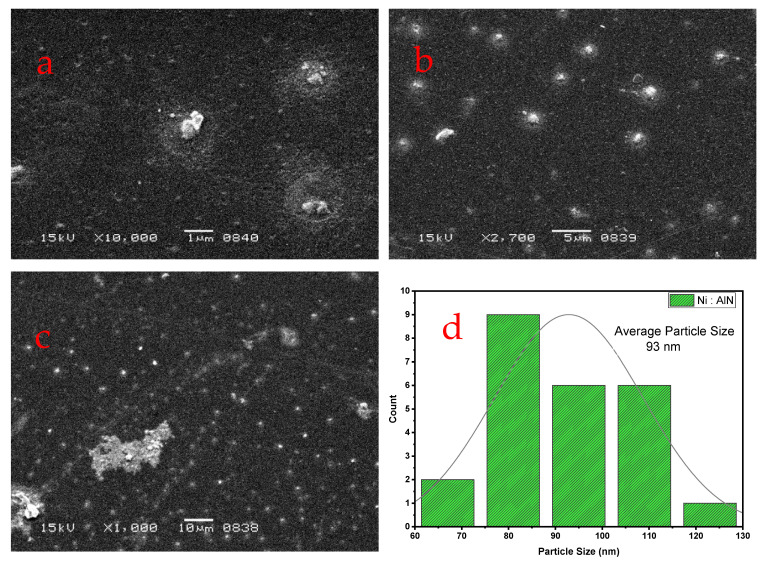
SEM of Ni: AlN thin films at different magnification powers: (**a**) 1 μm, (**b**) 5 μm, and (**c**) 10 μm). (**d**) Histogram for particle size.

**Figure 5:**
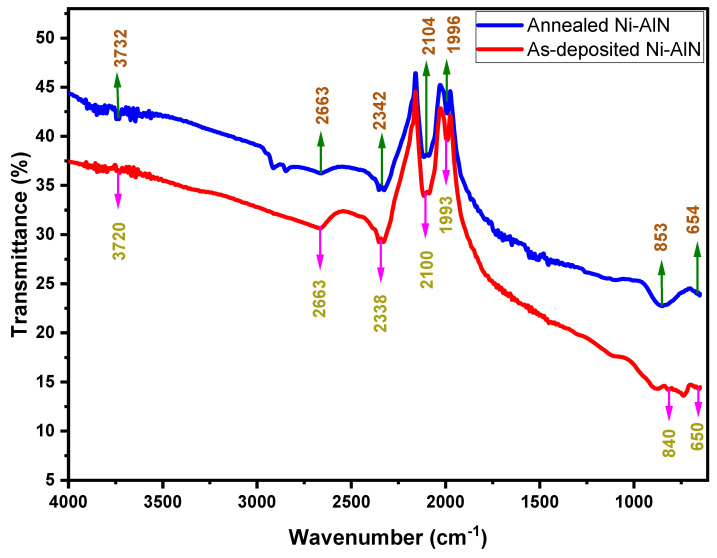
FTIR spectrum of the Ni: AIN thin film.

A 15 KV accelerating voltage was employed to perform SEM examination at various magnifications. Figure 4a–d depict an SEM picture of co-sputtered Ni-doped AlN thin films. It is shown that a homogeneous coating of AlN was applied to the Si substrate, with particles distributed evenly throughout the whole surface. The deposited sample exhibited grain-like spherical forms. Figure 5 depicts the particle size distribution illustrated by a histogram. During the investigation, it was determined that the surface of Ni: AlN thin films is made of agglomerated particles of uniform size, ~80–120 nm in diameter. Figure 4c illustrates the film picture at low magnification, whereas Figure 4a,b illustrate the film image at high magnification. Image J software was utilized to estimate the size of particles based on the 1 um magnification value. The smallest and largest particle sizes were determined to be 62 nm and 128 nm, respectively.

## 7. FTIR Analysis

Fourier transform infrared spectroscopy (FTIR) is an analytical technique used to determine the presence of chemical bonding, as well as the energy levels of existing interactions between atoms and chemical structures. The FTIR results presented in Figure 5 were obtained in the range of 4000–650 cm^–1^. In addition to the higher frequency region, the stretching vibrations of the O-H group were found to be responsible for the absorption bands at roughly 3720–3740 cm^−1^ [36]. The IR spectrum showed peaks around 1990–2100 cm^–1^, which could be attributed to the stretching modes of C-N and Al-N [37]. The presence of silicate in the substrate could explain the observation of the OH stretching mode. The existence of a modest absorption band at 853 cm^−1^ showed Si-O bonding, which had a negligible effect on the area under the curve but reduced the transmittance with irradiation. The stretching of transverse modes of wurtzite AlN structure resulted in peaks in the FTIR spectra at 650 cm^−1^ [38,39]. In addition, the FTIR spectrum demonstrated that the annealing temperature influenced the chemical structure of the deposited film. The increase in vibrational frequency of the deposited thin films, which shifted the peaks toward the higher wavenumber, was a clear cause of the change in the peak position and strength [40].

## 8. Photoluminescence Property

**Figure 6:**
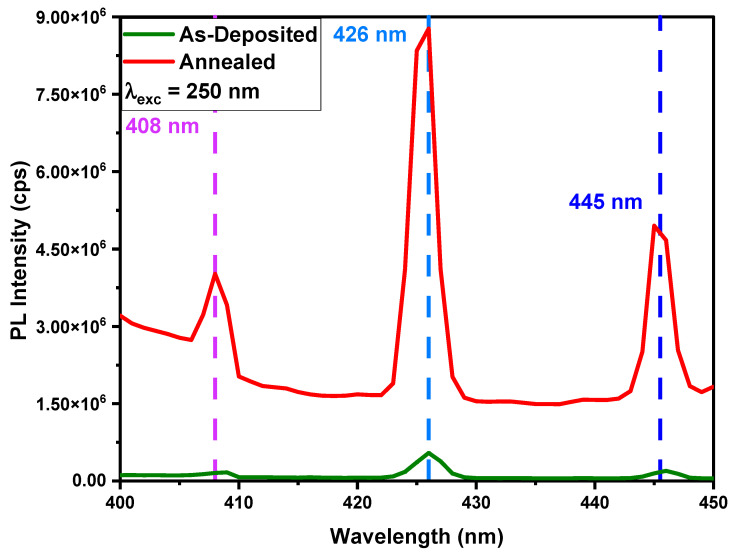
PL emission spectrum of as-deposited and annealed Ni-doped AlN thin films at an excitation wavelength of 250 nm.

Spectrofluorometer FS5, with a xenon arc lamp as an excitatory source, was used to examine a sample’s photoluminescence (PL). The PL emission spectra for both as-deposited and annealed thin films are illustrated in Figure 5 and Figure 6. It is apparent in the PL spectra that the majority of the emission peaks were recorded in the visible region. In the emission spectra, there were a vast number of PL bands with associated peak positions. The shift in PL band positions could be caused by changes in crystallinity and defects caused by doping. Because of increased crystallization inside the structure, the intensity of PL emissions was thought to rise after annealing. The higher the induced crystallization as a result of annealing, the greater the shift in peak intensity. The emission spectrum of deposited samples was obtained by fixing the excitation wavelength and scanning wavelengths from excitation mono-chromators in the desired range.

The spectrum in Figure 6 shows three strong peaks of varying strengths at different wavelengths in the spectral range of 400 nm to 450 nm, showing that the deposited layer was truly crystalline in nature. However, the bright-blue-colored emission peak at 426 nm had the highest intensity due to intra-center transitions in the deposited thin film. Since transition metals prefer to substitute cationic sites, Ni ions introduced into AlN films were thought to replace Al ions as nearest neighbors in a tetrahedral arrangement for four nitrogen atoms. As Ni atoms preferentially form Ni2+ ions to replace Al3+ ions, which can upset charge balance, nitrogen vacancies were spontaneously created to restore the charge equilibrium. Xiong et al. [20] reported a similar emission peak at 425 nm for the Ni-doped AlN thin films. Another study reported various emissions peaks for Cu-doped AlN monocrystalline powder [41] as an outcome of intra-center transitions and recombinations through interstitial and vacancy defects. Intra-center transitions correspond to those recombinations or the luminescence in which absorption, excitation, and emission occur within one of the same defects.

Herein, it was observed that after annealing, the strength of all peaks improved substantially; however, the intensity of the 426 nm peak did not increase proportionally. It increased much less than other peaks due to the existence of less Ni-related defects after annealing. The other type of process responsible for different peaks is the recombination mechanism, which involves two different types of defects. There are two options for this mechanism. The first type of luminescence is tunnel recombination luminescence. Tunneling happens from the excited state of defect to the ground state of defect with luminescence emission, or from the excited state of defect to the excited state of defect with some non-radiative emission. Other peaks may be caused by defects other than Ni impurities, including nitrogen vacancies, aluminum vacancies, interstitials, or oxygen-related defects.

**Figure 7:**
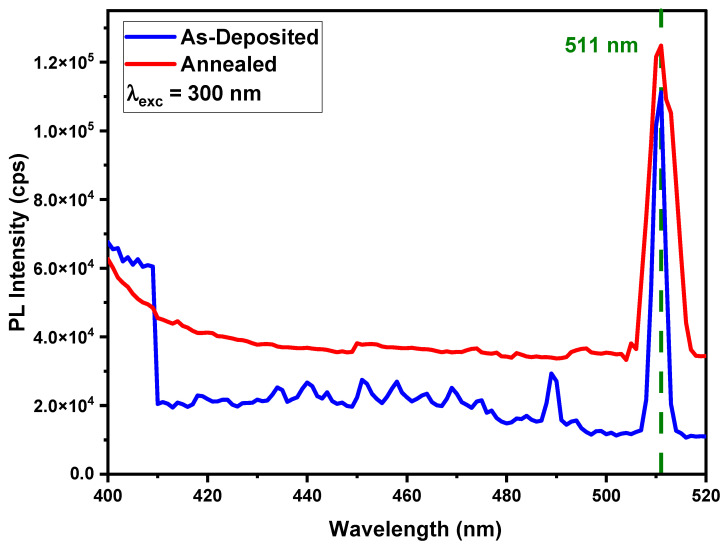
PL emission spectrum of as-deposited and annealed Ni-doped AlN thin films at an excitation of 300 nm.

Similarly, the PL emission spectra at 300 nm excitation wavelength revealed one sharp peak at 511 nm, as shown in Figure 7. A prominent green color emission peak at 511 nm was most intensely detected, which can be attributed to the recombination mechanism happening inside the material directly or indirectly. After annealing, the size of the crystallites became bigger and the number of defects improved, making the emission peak look better.

Hence, photoluminescence in AlN thin film was responsible for two mechanisms: (a) intra-center luminescence and (b) recombination ruminescence, which were observed by changing the concentration of different types of defects and excitation wavelengths.

## 9. Conclusions

This work effectively deposited Ni-AlN thin films via co-sputtering. The resulting thin film was thermally annealed at 1100 °C with continuous nitrogen flow to examine the influence of annealing on structural and photoluminescence characteristics. FTIR established material identification by presenting unique peaks in the fingerprint region. SEM investigation validated the morphology of the deposited films, which revealed a granular-like structure with an average particle size of 100 nm. The visible emission peaks produced by PL spectroscopy were quite strong and endorsed due to the intra-center transitions inside the Ni-associated defects. After annealing and adding Ni to the host material, improvements in crystal structure and photoluminescence properties were observed. The obtained results are exceedingly promising for optoelectronic applications, primarily in photovoltaic devices and lasers.

## Figures and Tables

**Table 1:** Crystallographic parameters of deposited Ni-doped AlN crystal structures.

Parameters	Values
Crystal system: Hexagonal	Space group: P63mc
a	3.1114 Å
b	3.1114 Å
c	4.9792 Å
α	90
β	90
γ	120

## Data Availability

Not applicable.

## Abbreviations

AlN: aluminum nitride; DC: direct current; RF: radio frequency; XRD: X-ray diffraction; FTIR: Fourier transform infrared spectroscopy; SEM: scanning electron microscopy; GaN: gallium nitride; ZnO: zinc oxide; Mn: manganese; Ni: nickel; Fe: iron; Cu: copper; Cr: chromium; NIR: near-infrared; PLD: pulsed laser deposition; CVD: chemical vapor deposition; MBE: molecular beam epitaxial; IPA: isopropyl alcohol; PL: photoluminescence.
